# Prevalence and Correlates of COVID-19 Vaccine Information on Family Medicine Practices’ Websites in the United States: Cross-sectional Website Content Analysis

**DOI:** 10.2196/38425

**Published:** 2022-11-17

**Authors:** Jonathan V Ackleh-Tingle, Natalie M Jordan, Udodirim N Onwubiko, Christina Chandra, Paige E Harton, Shelby T Rentmeester, Allison T Chamberlain

**Affiliations:** 1 Department of Epidemiology Rollins School of Public Health Emory University Atlanta, GA United States

**Keywords:** primary care, vaccine hesitancy, COVID-19, health communications, health information, health website, family practice, primary care, vaccine information, online health, health platform, online information

## Abstract

**Background:**

Primary care providers are regarded as trustworthy sources of information about COVID-19 vaccines. Although primary care practices often provide information about common medical and public health topics on their practice websites, little is known about whether they also provide information about COVID-19 vaccines on their practice websites.

**Objective:**

This study aimed to investigate the prevalence and correlates of COVID-19 vaccine information on family medicine practices’ website home pages in the United States.

**Methods:**

We used the Centers for Medicare and Medicaid National Provider Identifier records to create a sampling frame of all family medicine providers based in the United States, from which we constructed a nationally representative random sample of 964 family medicine providers. Between September 20 and October 8, 2021, we manually examined the practice websites of these providers and extracted data on the availability of COVID-19 vaccine information, and we implemented a 10% cross-review quality control measure to resolve discordances in data abstraction. We estimated the prevalence of COVID-19 vaccine information on practice websites and website home pages and used Poisson regression with robust error variances to estimate crude and adjusted prevalence ratios for correlates of COVID-19 vaccine information, including practice size, practice region, university affiliation, and presence of information about seasonal influenza vaccines. Additionally, we performed sensitivity analyses to account for multiple comparisons.

**Results:**

Of the 964 included family medicine practices, most (n=509, 52.8%) had ≥10 distinct locations, were unaffiliated with a university (n=838, 87.2%), and mentioned seasonal influenza vaccines on their websites (n=540, 56.1%). In total, 550 (57.1%) practices mentioned COVID-19 vaccines on their practices’ website home page, specifically, and 726 (75.3%) mentioned COVID-19 vaccines anywhere on their practice website. As practice size increased, the likelihood of finding COVID-19 vaccine information on the home page increased (n=66, 27.7% among single-location practices, n=114, 52.5% among practices with 2-9 locations, n=66, 56.4% among practices with 10-19 locations, and n=304, 77.6% among practices with 20 or more locations, *P*<.001 for trend). Compared to clinics in the Northeast, those in the West and Midwest United States had a similar prevalence of COVID-19 vaccine information on website home pages, but clinics in the south had a lower prevalence (adjusted prevalence ratio 0.8, 95% CI 0.7 to 1.0; *P*=.02). Our results were largely unchanged in sensitivity analyses accounting for multiple comparisons.

**Conclusions:**

Given the ongoing COVID-19 pandemic, primary care practitioners who promote and provide vaccines should strongly consider utilizing their existing practice websites to share COVID-19 vaccine information. These existing platforms have the potential to serve as an extension of providers’ influence on established and prospective patients who search the internet for information about COVID-19 vaccines.

## Introduction

As of July 2022, at least 1 million Americans have died from COVID-19, although official counts may underestimate total attributable deaths [1,2]. While 67% of the total population has been fully vaccinated against COVID-19 [1], achieving community protection requires a coverage of at least 80% [3]. However, gains in vaccination coverage within the United States have slowed, in part owing to vaccine hesitancy, which is defined as a “delay in acceptance or refusal of [COVID-19] vaccination despite availability of vaccination services” [4,5].

COVID-19 vaccine hesitancy is a well-established infodemiological problem in public health, resulting from a complex interplay among many factors, including, but not limited to, systemic racism, political ideology, intentional and unintentional spread of misinformation, social media networks and behaviors, and skepticism of the scientific community [6-14]. While longer-term solutions to COVID-19 vaccine hesitancy, specifically—as well as hesitancy toward other vaccines, more generally—will require targeting these systems-level causes, shorter-term interventions can also improve COVID-19 vaccine uptake by addressing downstream factors that influence vaccine intent at the level of clinics and individuals, such as individuals’ health literacy and vaccine proponents’ trustworthiness [15-17]. In the early stages of the COVID-19 pandemic, the US Centers for Disease Control and Prevention identified many of these factors as targets of their national campaign, “Vaccinate with Confidence: Strategy to Reinforce Confidence in COVID-19 Vaccines” [17]. In particular, the Vaccinate with Confidence campaign emphasizes the importance of “effective messages delivered by trusted messengers,” in which physicians and other health care professionals tailor narratives about COVID-19 vaccines to the unique needs of their particular patient populations [17].

Primary care providers (PCPs) are consistently regarded as trusted messengers for vaccine-related information and are well-qualified to communicate effectively to overcome hesitancy [17-19]. Enhancing PCPs’ web-based promotion of COVID-19 vaccines through their existing practice websites may allow them to reach individuals seeking COVID-19 vaccine information on the internet. Patients already visit these websites to access logistical information such as practice address, telephone number, and patient portals. However, it is unclear whether PCPs use their websites to communicate information about COVID-19 vaccines. The aim of this paper was to describe the prevalence and correlates of the provision of COVID-19 vaccine information on family medicine practices’ websites in the United States.

## Methods

### Overview

Using the National Provider Identifier records of the Centers for Medicare and Medicaid Services’ National Plan and Provider Enumeration System, we created a sampling frame of 136,531 providers who indicated “family medicine” as their primary taxonomy code and were practicing in the United States as of September 12, 2021. Using this sampling frame, we selected an overall target sample size of 1000 unique websites, maintaining convention with nationwide surveys and polls [20], as well as similar exploratory analyses for which no a priori power calculations exist [21]. Ensuring a nationally representative sample, the state-level target sample size was determined on the basis of the proportion of the total number of family medicine providers listed for that state. For example, if a state had 1365 providers listed in the sampling frame (1% of 136,531), that state’s target sample size in our sample would be 10 websites (1% of 1000). For each state, a random number generator assigned each practitioner a number, and then the team sorted the observations by the random number and selected the top *n* observations (*n*=state-level target sample size).

Five data abstractors searched internet browsers from September 20 to October 8, 2021, to identify sampled providers’ practice websites. Websites were excluded if the practice (1) did not have an apparent focus on family medicine or primary care, (2) was located in a different state than that listed in the National Provider Identifier database, (3) was a duplicate, (4) had a nonfunctional website link, (5) used only social media pages (eg, Facebook), or (6) was permanently closed. Websites affiliated with the military or court-mandated health care systems were also excluded owing to their varying approaches to vaccine policies, which may have included messaging that the general public was not exposed to stringent vaccine mandates that were not applicable to the general public, or limited autonomy to shape vaccine messaging at the level of individual clinics and providers. For data quality control, the team cross-reviewed 10% of the websites, resolving any discordances in data extraction.

Our primary outcome was a mention of COVID-19 vaccines on the practices’ website home page, specifically. Secondary outcomes included a mention of COVID-19 vaccines anywhere on the website, evidence that a clinic provided COVID-19 vaccines on site, provision of tailored vaccine information, explicit mention of full Food and Drug Administration (FDA) approval of the Pfizer-BioNTech COVID-19 vaccine (the only fully FDA-approved COVID-19 vaccine at the time of data collection [22,23]), and provision of a frequently asked question (FAQ) section about COVID-19 vaccines. “Tailored vaccine information” was defined as COVID-19 vaccine information thoughtfully curated or presented in a patient-centered manner or from the perspective of the providers in that clinic specifically. For example, an explicit mention of the patient populations served by the clinic or explicit acknowledgment of common needs and concerns of the clinic’s population would illustrate tailoring of standard vaccine information. Exploratory independent variables included the number of clinic locations, affiliation with a large hospital or managed health care system (conglomerate affiliation), university affiliation, mention of seasonal influenza vaccination, and US Census Bureau–defined geographic region within the United States [24]. We specifically explored the promotion of seasonal influenza vaccines because we sought to verify whether providers adopted similar policies of promoting another vaccine with well-established vaccine hesitancy in the general public [25].

We estimated the period prevalence of all outcomes. We estimated period prevalence, rather than point prevalence, because our data collection process was carried out over a period of several weeks rather than at a single time point. We reported unstratified results, as well as results stratified by the number of clinic locations. Cochran-Armitage tests for trend were used to identify statistically significant differences across strata of the number of clinic locations. The Cochran-Armitage test for trend is appropriate for assessing whether data in a contingency table of dimensions 2×*C*, where *C* is an ordinal variable with >2 levels, differ between the 2 groups [26,27]. We verified that all expected cell counts in these 2×*C* tables were *n*>5.

Independent variables associated with mentioning COVID-19 vaccines on the website home page were assessed using modified Poisson regression models with robust error variances. In these analyses, our primary and secondary outcomes were each modeled as binary indicator outcome variables, which were set to 0 when a practice website did not have the attribute to 1 when the practice did. Modified Poisson regression is appropriate when estimating the prevalence or risk (including prevalence and risk ratios) of a high-prevalence, binary outcome [28,29]. We assumed that all observations were independent. Given the exploratory nature of our regression analyses, we also assumed no interaction between our independent variables, and we assumed that each variable had a linear association with the outcome on the log scale. Finally, we conducted a sensitivity analysis for these Poisson regression models in which *P* values were adjusted for multiple comparisons [30-33].

Statistical analyses were performed using SAS (version 9.4; SAS Institute Inc). All data analyzed in this study are included in Multimedia Appendices 1 and 2.

### Ethical Considerations

There were no human participants in this study, and all data were collected via publicly available internet searches. Therefore, this study did not require ethics approval.

## Results

After applying the exclusion criteria, 964 unique practice websites were included in the analytic sample. Practices typically had ≥10 locations (n=509, 52.8%), were affiliated with a conglomerate (n=663, 68.2%), were unaffiliated with a university (n=838, 87.2%), and mentioned seasonal influenza vaccines on their website (n=540, 56.1%; Table 1).

Between September 20 and October 8, 2021, overall, 550 (57.1%) websites mentioned COVID-19 vaccines on their home page, and 726 (75.3%) mentioned them somewhere on their website (Table 2). Additionally, 580 (60.2%) tailored the content provided, 426 (44.2%) provided a COVID-19 vaccine–focused FAQ section, and 199 (20.6%) explicitly mentioned the Pfizer BioNTech vaccine’s full FDA approval.

Mentioning COVID-19 vaccines on home pages ranged from 28% among single-location practices to 78% among ≥20-location practices (*P*<.001 for trend). Similar statistically and clinically significant trends were observed between the number of clinic locations and all other outcomes (Table 2).

After adjusting for university affiliation, mention of influenza vaccines, and geographic region, the prevalence of mentioning COVID-19 vaccines on the home page was 1.7 (95% CI 1.4 to 2.2), 1.7 (95% CI 1.3 to 2.2), and 2.3 (95% CI 1.8 to 2.8) times higher among clinics with 2-9, 10-19, or ≥20 locations, respectively, than for clinics with only 1 location (Table 3). Compared to websites from Northeastern practices, those from the West and Midwest United States had comparable outcome prevalence; practices in the south had a lower prevalence (adjusted prevalence ratio 0.8, 95% CI 0.7 to 1.0). Sensitivity analyses, in which Poisson regression *P* values were adjusted for multiple comparisons, yielded similar statistical results (Multimedia Appendix 2, Table S1).

**Table 1:** Characteristics of family medicine practices in the United States included in our fall 2021 COVID-19 website content review.

Practice characteristics			Frequency (n=964), n (%)
**Practice locations**			
	1	238 (24.7)	
	2-9	217 (22.5)	
	10-19	117 (12.1)	
	≥20	392 (40.7)	
**Practice affiliated with a large hospital or managed health care system (conglomerate)**			
	Yes	663 (68.2)	
	No	301 (31.2)	
**Practice affiliated with a university**			
	Yes	123 (12.8)	
	No	838 (87.2)	
**Practice website mentions seasonal influenza vaccination**			
	Yes	540 (56.1)	
	No	423 (43.9)	
**Translations to other languages available on website**			
	Yes	266 (27.6)	
	No	698 (72.4)	
**US region^a^**			
	Midwest	243 (25.2)	
	Northeast	145 (15.0)	
	South	333 (34.5)	
	West	243 (25.2)	

^a^US regions: Midwest (Iowa, Illinois, Indiana, Kansas, Michigan, Minnesota, Missouri, North Dakota, Nebraska, Ohio, South Dakota, and Wisconsin), northeast (Connecticut, Massachusetts, Maine, New Hampshire, New Jersey, New York, Pennsylvania, Rhode Island, and Vermont), south (Alabama, Arkansas, District of Columbia, Delaware, Florida, Georgia, Kentucky, Louisiana, Maryland, Mississippi, North Carolina, Oklahoma, South Carolina, Tennessee, Texas, Virginia, and West Virginia), and west (Alaska, Arizona, California, Colorado, Hawaii, Idaho, Montana, New Mexico, Nevada, Oregon, Utah, Washington, and Wyoming).

**Table 2:** COVID-19 vaccine–related information on family medicine practice websites in the United States included in our fall 2021 COVID-19 website content review.

		Total, n (%)	Practice locations, n (%)				*P* value^a^	
			1	2-9	10-19	≥20		
**Mentions the COVID-19 vaccine on the home page**								<.001
	Yes	550 (57.1)	66 (27.7)	114 (52.5)	66 (56.4)	304 (77.6)		
	No	414 (42.9)	172 (72.3)	103 (47.5)	51 (43.6)	88 (22.4)		
**Mentions the COVID-19 vaccine mentioned anywhere on the website**								<.001
	Yes	726 (75.3)	98 (41.2)	158 (72.8)	97 (82.9)	373 (95.2)		
	No	238 (24.7)	140 (58.8)	59 (27.2)	20 (17.1)	19 (4.9)		
**Provides tailored information on COVID-19 vaccines^b^**								<.001
	Yes	580 (60.2)	59 (24.8)	114 (52.5)	71 (60.7)	336 (85.7)		
	No	384 (39.8)	179 (75.2)	103 (47.5)	46 (39.3)	56 (14.3)		
**Mentions Food and Drug Administration approval of the COVID-19 vaccine^c^**								<.001
	Yes	199 (20.6)	15 (6.3)	22 (10.1)	24 (20.5)	138 (35.2)		
	No	765 (79.4)	223 (93.7)	195 (89.9)	93 (79.5)	254 (64.8)		
**Mentions the booster or third dose of the vaccine**								<.001
	Yes	400 (41.5)	28 (11.8)	67 (30.9)	55 (47.0)	250 (63.8)		
	No	564 (58.5)	210 (88.2)	150 (69.1)	62 (53.0)	142 (36.2)		
**Has information on COVID-19 vaccine eligibility criteria**								<.001
	Yes	530 (55.0)	51 (21.4)	101 (46.5)	66 (56.4)	312 (79.6)		
	No	434 (45.0)	187 (78.6)	116 (53.5)	51 (43.6)	80 (20.4)		
**Has a “frequently asked questions” section on COVID-19 vaccines on the website**								<.001
	Yes	426 (44.2)	31 (13.0)	62 (28.6)	48 (41.0)	285 (72.7)		
	No	538 (55.8)	207 (87.0)	155 (71.4)	69 (59.0)	107 (27.3)		
**Nature of the website’s language regarding COVID-19 vaccination**								<.001
	No mention	245 (25.4)	142 (59.7)	64 (29.5)	22 (19.0)	17 (4.3)		
	Neutral	172 (17.9)	37 (15.5)	51 (23.5)	25 (21.6)	59 (15.1)		
	Supportive	546 (56.7)	59 (24.8)	102 (47.0)	69 (59.5)	316 (80.6)		

^a^Cochran-Armitage trend test.

^b^Tailored information described as curated information indicating an effort from the practice to explain and endorse the COVID-19 vaccines.

^c^The website explicitly mentioned the approval of the COVID-19 vaccine (Pfizer’s Comirnaty approved on August 23, 2021) by the US Food and Drug Administration anywhere on the website.

**Table 3:** Unadjusted and adjusted prevalence ratios of whether a family medicine practice mentioned the COVID-19 vaccine on their website home page.

		Mentioned the COVID-19 vaccine on the home page, n (%)	Unadjusted prevalence ratio			Adjusted prevalence ratio^a^	
			Ratio (95% CI)	*P* value	Ratio (95% CI)		*P* value
**Number of locations**							
	1	66 (27.7)	Reference	—^b^	Reference		—
	2-9	114 (52.5)	1.89 (1.49-2.41)	<.001	1.73 (1.37-2.20)		<.001
	10-19	66 (56.4)	2.03 (1.57-2.64)	<.001	1.73 (1.34-2.23)		<.001
	≥20	304 (77.6)	2.80 (2.26-3.46)	<.001	2.25 (1.81-2.79)		<.001
**US region^c^**							
	Northeast	93 (64.1)	Reference	—	Reference		—
	Midwest	148 (60.9)	0.95 (0.81-1.11)	.52	0.96 (0.84-1.10)		.57
	South	166 (49.9)	0.78 (0.66-0.91)	.002	0.84 (0.73-0.97)		.02
	West	143 (58.9)	0.92 (0.78-1.08)	.29	0.99 (0.86-1.14)		.86
University-affiliated^d^		94 (76.4)	1.41 (1.26-1.59)	<.001	1.15 (1.03-1.29)		.01
Mentioned influenza vaccination^d^		394 (73.0)	1.99 (1.74-2.28)	<.001	1.62 (1.41-1.86)		<.001

^a^Models adjusted for the number of locations, region, university affiliation, and mention of seasonal influenza vaccines.

^b^—: not applicable.

^c^US regions: Midwest (Iowa, Illinois, Indiana, Kansas, Michigan, Minnesota, Missouri, North Dakota, Nebraska, Ohio, South Dakota, and Wisconsin), northeast (Connecticut, Massachusetts, Maine, New Hampshire, New Jersey, New York, Pennsylvania, Rhode Island, and Vermont), south (Alabama, Arkansas, District of Columbia, Delaware, Florida, Georgia, Kentucky, Louisiana, Maryland, Mississippi, North Carolina, Oklahoma, South Carolina, Tennessee, Texas, Virginia, and West Virginia), and west (Alaska, Arizona, California, Colorado, Hawaii, Idaho, Montana, New Mexico, Nevada, Oregon, Utah, Washington, and Wyoming).

^d^Comparing “Yes” versus “No” responses (reference).

## Discussion

### Principal Findings

As revealed in this analysis, at the time of data collection, not all family medicine practices were maximizing their existing practice websites for COVID-19 vaccine promotion. As of October 2021, nearly half of the websites examined by our research team did not mention COVID-19 vaccines on their home page. In addition, only one-fifth of them mentioned full FDA approval of the Pfizer vaccine, a regulatory accomplishment that many postulated would increase vaccine confidence and uptake [34]. Although medical professionals may have missed the opportunity to amplify this milestone on their practices’ websites, future milestones may present similar opportunities, including full FDA approval of vaccines for young children and approval of additional strain-specific booster vaccines.

It is critical for PCPs, as trusted sources of vaccine-related information, to optimize communication with the general public [18,19]. PCPs can accomplish medical and public health goals concurrently by considering how their website can serve as a web-based extension of the tailored messaging they often use inside the clinic. Furthermore, given the potential for novel SARS-CoV-2 variants (eg, the B1 variant) and future infection surges, it is increasingly important for vaccine providers to be cognizant of where and how to make thoughtful use of their existing practice websites for additional vaccine promotion.

How vaccine content is described is also important. With only 60% of included websites providing tailored content, there is additional room to translate the tailored, values-based messaging often used inside clinics to the clinic’s internet domain [35,36]. If curated appropriately on the home page, a practice website can quickly convey the priorities and values of the practice as they pertain to vaccines.

Our analysis also revealed that as practice size increased, the presence of COVID-19 vaccine information also increased. Although our analysis was not designed to determine the cause of any association between practice size and web-based vaccine promotion, we hypothesize that this finding reflects that larger, well-resourced practices have greater means to update their websites. This would signal an opportunity for entities such as national professional societies to develop guidance to help less-resourced practices provide accurate and specialty-specific information on the internet.

Finally, we observed that compared to northeastern clinics, southern clinics were less likely to mention COVID-19 vaccines on their practices’ website home pages. This finding emerges amid evidence that southern states continue to have COVID-19 vaccination coverage below the national average [37]. Given the lower baseline rates of COVID-19 information on the home pages of websites of southern clinics, we hypothesize that in the future, concerted efforts to increase COVID-19 vaccine information on practices’ websites may have the highest overall impact in this region.

### Strengths and Limitations

The strengths of our study include a large, nationwide sampling frame of family medicine practitioners to yield a sample distributed proportionally across all 50 states. Furthermore, our team implemented a 10% cross-checking measure to reduce data entry errors and improve internal validity.

Our study also has limitations. As our study is exploratory in nature, it is not designed to assess the causal impact of web-based information on the COVID-19 vaccine on the population uptake of these vaccines. In addition, this study’s cross-sectional design does not reflect whether and how clinics change their website content over time. Finally, our analytic sample did not include other relevant provider types (eg, pediatricians) who may differ in approaches to provision of vaccine content on their websites.

### Conclusions

During a pandemic, it is vital for medical providers to utilize all means to promote vaccination when safe and effective vaccines are available. With many PCPs already hosting websites, there is little reason not to utilize this platform to reach patients who are eager to understand their trusted provider’s views.

## Appendix

Multimedia Appendix 1 This includes our raw and analysis-ready datasets.

Multimedia Appendix 2 This is our sensitivity analysis to account for multiple testing.

## Abbreviations

FDA: Food and Drug Administration
PCP: primary care provider
