# Supplementary material for: Prevalence and Correlates of COVID-19 Vaccine Information on Family Medicine Practices’ Websites in the United States: Cross-sectional Website Content Analysis
Source: JMIR Form Res. 2022 Nov 17;6(11):e38425. doi: 10.2196/38425 (PMC9671487; doi:10.2196/38425)
Supplement: Multimedia Appendix 2 [file formative_v6i11e38425_app2.docx]

Supplement 1

The goal of our regression analyses was to identify practice-level predictors of mentioning COVID-19 vaccine information on primary care practice websites. In recognition of the increased risk of committing a Type 1 error due to repeated tests of independent predictor variables [S1], we conducted a sensitivity analysis to adjust the p-values from our regressions.

Using PROC MULTTEST in SAS 9.4 [S2], we imported the original p-values from our univariable and multivariable regressions as presented in Table 3, and we used these raw p-values to calculate adjusted p-values using the step-down Bonferroni and false discovery rate approaches [S3].

The step-down Bonferroni approach derives from a family of techniques to control the family-wise error rate, or the overall Type 1 error rate, for a set of statistical tests. In contrast to the original Bonferroni approach to adjusting for multiple comparisons, which is known to be very conservative in prioritizing avoidance of Type 1 errors at the expense of lower statistical power, the step-down Bonferroni approach sequentially determines adjusted p-values for all raw p-values considered, which preserves some degree of statistical power [S3]. In contrast to Bonferroni-style adjustments for multiple testing, which control the probability of incorrectly rejecting a truly null hypothesis, a false discovery rate-oriented approach controls the probability of truly null hypotheses among tests deemed statistically significant. It is a well-established adjustment approach in genomic studies, and it is also known to preserve statistical power [S3, S4].

Generally, our p-values obtained without adjustment for multiple comparisons were similar to those obtained with adjustment. Number of clinic locations remained highly statistically significant regardless of the p-value calculation method, as was whether a clinic mentioned routine influenza vaccinations on their website. Geographic region had similar results across p-value methods, although the statistical significance of a clinic’s presence in the South was attenuated slightly in the multivariable regression model when using the Step-Down Bonferroni approach. University affiliation remained statistically significant using the false discovery rate adjustment method but was no longer statistically significant when using the Step-Down Bonferroni method (p=.07).

Taken together, these results are reassuring that reported results in Table 3 are not simply a statistical artefact of multiple comparisons.

Table S1. Univariable and multivariable regression p-values for predictors of whether a family medicine practice mentioned the COVID-19 vaccine on their website homepage, with p-value adjustments for the problem of multiple comparisons.

|  | Mentioned COVID-19 vaccine on homepage  N (%) | Univariable regressions | | | Multivariable regression^a^ | | |
| --- | --- | --- | --- | --- | --- | --- | --- |
|  |  | P-value adjustment technique | | | P-value adjustment technique | | |
|  |  | None^d^ | SDB^e^ | FDR^f^ | None^d^ | SDB^e^ | FDR^f^ |
| **Number of locations** |  |  |  |  |  |  |  |
| 1 | 66 (27.7) | Ref | Ref | Ref | Ref | Ref | Ref |
| 2-9 | 114 (52.5) | <.001 | <.001 | <.001 | <.001 | <.001 | <.001 |
| 10-19 | 66 (56.4) | <.001 | <.001 | <.001 | <.001 | <.001 | <.001 |
| 20+ | 304 (77.6) | <.001 | <.001 | <.001 | <.001 | <.001 | <.001 |
| **US Region^b^** |  |  |  |  |  |  |  |
| Northeast | 93 (64.1) | Ref | Ref | Ref | Ref | Ref | Ref |
| Midwest | 148 (60.9) | .52 | >.99 | .60 | .57 | >.99 | .61 |
| South | 166 (49.9) | .002 | .02 | .004 | .02 | .10 | .03 |
| West | 143 (58.9) | .29 | >.99 | .36 | .86 | >.99 | .86 |
| **University-affiliated^c^** | 94 (76.4) | <.001 | <.001 | <.001 | .01 | .07 | .02 |
| **Mentioned influenza vaccination^c^** | 394 (73.0) | <.001 | <.001 | <.001 | <.001 | <.001 | <.001 |

^a^Model covariates including: number of locations, region, university affiliation, and mention of seasonal influenza vaccine.

^b^US Regions: **Midwest** (Iowa, Illinois, Indiana, Kansas, Michigan, Minnesota, Missouri, North Dakota, Nebraska, Ohio, South Dakota, Wisconsin); **Northeast** (Connecticut, Massachusetts, Maine, New Hampshire, New Jersey, New York, Pennsylvania, Rhode Island, Vermont); **South** (Alabama, Arkansas, District of Columbia, Delaware, Florida, Georgia, Kentucky, Louisiana, Maryland, Mississippi, North Carolina, Oklahoma, South Carolina, Tennessee, Texas, Virginia, West Virginia); **West** (Alaska, Arizona, California, Colorado, Hawaii, Idaho, Montana, New Mexico, Nevada, Oregon, Utah, Washington, Wyoming).

^c^Comparing “Yes” vs. “No” (reference).

^d^No p-value adjustments made; p-values come directly from Poisson regression output.

^e^P-values adjusted using the Step-Down Bonferroni approach.

^f^P-values adjusted using the False Discovery Rate approach.

## Supplement References

S1. Feise RJ. Do multiple outcome measures require p-value adjustment? BMC Med Res Methodol. 2002;2:8. doi:10.1186/1471-2288-2-8.

S2. SAS Help Center: PROC MULTTEST Statement. Accessed August 2, 2022. https://documentation.sas.com/doc/en/pgmsascdc/9.4_3.3/statug/statug_multtest_syntax01.htm.

S3. SAS Help Center: p-Value Adjustments. Accessed August 2, 2022. https://documentation.sas.com/doc/en/pgmsascdc/9.4_3.3/statug/statug_multtest_details11.htm.

S4. Glickman ME, Rao SR, Schultz MR. False discovery rate control is a recommended alternative to Bonferroni-type adjustments in health studies. J Clin Epidemiol. 2014;67(8):850-857. doi:10.1016/j.jclinepi.2014.03.012.
